# Hypothermia in preterm infants admitted to low-resource neonatal units in northern Nigeria: an observational study of occurrence and risk factors

**DOI:** 10.1186/s12887-024-04960-3

**Published:** 2024-07-24

**Authors:** Usman Abiola Sanni, Fatima Usman, Taofik Oluwaseun Ogunkunle, Adamu Sa’idu Adamu, Audu Isah Lamidi, Taslim Olatunde Lawal, Surajudeen Oyeleke Bello, Aliyu Mamman Na’uzo, Tajudeen Lanre Ibrahim, Nyirimanzi Naphtal, Sa’adatu Shehu, Abdullahi Jibrin, Zubaida Ladan Farouk, Muhammad Faruk Bashir, Idris Abiodun Adedeji, Mohammed Abdulsalam, Yakubu Abdullahi, Abdulazeez Imam

**Affiliations:** 1 Partners in Health, Koidu, Kono Sierra Leone; 2 Department of Pediatrics, Koidu Government Hospital, Koidu, Kono Sierra Leone; 3 Aminu Kano Teaching Hospital, Kano, Nigeria; 4 Bayero University, Kano, Nigeria; 5 Dalhatu Araf Specialist Hospital, Lafia, Nigeria; 6 Abubakar Tafawa Balewa University Teaching Hospital, Bauchi, Nigeria; 7 Abubakar Tafawa Balewa University, Bauchi, Nigeria; 8 Federal University of Health Sciences, Azare, Nigeria; 9 Federal Medical Center, Birnin Kebbi, Nigeria; 10 Health Systems Collaborative, Nuffield Department of Medicine, University of Oxford, S Parks Rd, Oxford, OX1 3SY UK

**Keywords:** Neonatal hypothermia, Risk factors, Northern Nigeria, Prematurity

## Abstract

**Background:**

Hypothermia is an important cause of morbidity and mortality among preterm and low-birth-weight neonates. In resource-constrained settings, limited referral infrastructure and technologies for temperature control potentiate preterm hypothermia. While there is some documentation on point-of-admission hypothermia from single center studies, there are limited multicenter studies on the occurrence of hypothermia among preterm infants in resource-limited-settings. Therefore, we conducted a multicenter study to determine the prevalence and risk factors for hypothermia at the time of admission and during the first 72 h after admission in northern Nigeria.

**Method:**

We carried out a prospective cohort study on preterm infants admitted to four referral hospitals in northern Nigerian between August 2020 and July 2021. We documented temperature measurements at admission and the lowest and highest temperatures in the first 72 h after admission. We also collected individual baby-level data on sociodemographic and perinatal history data. We used the World Health Organization classification of hypothermia to classify the babies’ temperatures into mild, moderate, and severe hypothermia. Poisson regression analysis was used to identify risk factors for moderate-severe hypothermia.

**Results:**

Of the 933 preterm infants enrolled, 682 (72.9%) had hypothermia at admission although the prevalence of hypothermia varied across the four hospitals. During the first 24 h after admission, 7 out of every 10 babies developed hypothermia. By 72 h after admission, between 10 and 40% of preterm infants across the 4 hospitals had at least one episode of moderate hypothermia. Gestational age (OR = 0.86; CI = 0.82–0.91), birth weight (OR = 8.11; CI = 2.87–22.91), presence of a skilled birth attendant at delivery (OR = 0.53; CI = 0.29–0.95), place of delivery (OR = 1.94 CI = 1.13–3.33) and resuscitation at birth (OR = 1.79; CI = 1.27–2.53) were significant risk factors associated with hypothermia.

**Conclusion:**

The prevalence of admission hypothermia in preterm infants is high and hypothermia is associated with low-birth-weight, place of delivery and presence of skilled birth attendant. The prevalence of hypothermia while in care is also high and this has important implications for patient safety and quality of patient care. Referral services for preterm infants need to be developed while hospitals need to be better equipped to maintain the temperatures of admitted small and sick newborns.

## Background

Globally, hypothermia continues to affect a significant proportion of newborns with devastating consequences for their health and survival [1, 2].Warm tropical countries are no exception to this despite their ambient temperatures [1, 3].The prevalence of hypothermia is especially high among preterm and very low birth weight infants due to their reduced amount of brown fat and subcutaneous fat, in addition to a poorly developed response to thermal stress [4, 5].

Preterm infants are prone to hypothermia at any point during contact with the healthcare system. This can occur at admission or during the hospital stay [6] While admission hypothermia largely reflects prehospital transfer conditions, hypothermia in hospital care reflects either ineffective temperature care or the occurrence of newborn complications such as neonatal infection which can be associated with hypothermia [7].

Studies that have focused on preterm admission hypothermia have reported varying incidences between 31% and 87.9% [3, 8–10] Additionally, studies have documented a link between admission hypothermia and poor neonatal outcomes. A multicenter study from Brazil reported that admission hypothermia was significantly associated with early neonatal death irrespective of hospital performance [11]. Additionally, a study from Ethiopia showed that mortality increased with decreasing admission temperature among preterm babies [3]. High rates of complications such as severe neurological injury, severe retinopathy of prematurity (ROP), necrotizing enterocolitis (NEC), and bronchopulmonary dysplasia (BPD) were also found to be associated with admission hypothermia [1, 2]. Admission hypothermia is thus associated with significant preterm infant morbidity and mortality. Clinical studies have demonstrated the effectiveness of simple and relatively inexpensive temperature management strategies such as plastic wrapping without drying, the use of trans-warmer mattresses, kangaroo mother care and chemical mattresses [13–17]. Despite these findings, little evidence from Low and Middle Income Countries (LMIC) has shown that a significant proportion of preterm infants are not kept warm at birth due to a lack of resources and expertise to manage these infants [18, 19]. In addition, in resource-constrained settings, many high-risk deliveries (including those of preterm mothers) occur outside of tertiary obstetric facilities and neonatal transport services are severely limited [20]. Studies on hypothermia and its associated risk factors in such under sourced settings are important for improving the understanding of these problems in such settings and facilitating the design of suitable interventions to decrease the incidence of hypothermia.

Northern Nigeria has been observed to lag behind in terms of healthcare infrastructure, neonatal survival and other child survival indices and might be representative of typical low resource setting. According to the 2018 National Demographic and Health Survey (NDHS), the neonatal mortality rate in northern Nigeria was 32–47/ 1,000 live births whereas in Southern Nigeria, the neonatal mortality rate was 27–31/ 1,000 live births [21]. Northern Nigeria therefore represents a good setting to study preterm infants in under-resourced neonatal units. Hence, we carried out a prospective multicenter cohort study on hypothermia in preterm infants admitted to low-resource neonatal units. This builds on the previous studies that have been mostly single center cross-sectional studies with smaller sample sizes. In this study, we investigated the occurrence and associated risk factors for hypothermia in four tertiary level hospitals across northern Nigeria with staff and wider resource constraints.

## Subjects and methods

### Study design and setting

We conducted a multisite prospective cohort study of preterm infants in the neonatal units of four tertiary referral hospital facilities in northern Nigeria - Dalhatu Araf Specialist Hospital (DASH), Federal Medical Centre (FMC) Birnin-Kebbi, Aminu Kano Teaching Hospital (AKTH) and the Abubakar Tafa Balewa University Teaching Hospital (ATBUTH). Each of these neonatal units provides newborn care 24 h a day and 7 days a week to communities within their local environs and serves as referral centers for neighboring hospitals, where neonatal care is either unavailable or needed. These newborn units experience challenges related to human resources, space, and medical equipment common to other low-resource newborn unit settings. Table 1 provides a summary of these facilities.

**Table 1:** Summary of health facilities

Variable	AKTH	ATBUTH	FMC Birnin Kebbi	DASH
Number of newborn beds	34	27	20	35
Average yearly admissions (including term infants)	1672	1066	615	809
Average number of nurses on a day shift	4	4	5	3
Average number of nurses per night shift	3	2	3	3
Presence of a separate KMC ward	No	No	No	No
Current number of medical officers and residents working in SCBU	2	3	2	3
Number of Pediatricians working in SCBU	4	1	2	1
Number of functional incubators	20	4	6	4
Number of functional radiant warmers	2	3	2	1

SCBU = Special Care Baby Unit

### Study population

Our study population consisted of preterm infants who were less than 24 h old and were admitted for neonatal care in the participating newborn units. These infants were either born in each of the participating facilities and then admitted to our units or were born outside the facilities but were referred to us within 24 h after delivery.

### Data collection

Research assistants administered a structured proforma to mothers and caregivers of eligible preterm infants who consented to participate in the study and demographic, maternal, delivery and relevant clinical data were collected. All eligible babies alongside the non-eligible ones received the appropriate care that included clinical history and vital sign measurements from the admitting team. These data were transcribed into our proforma by trained research assistants. When data were not available for selected fields, caregivers were asked if they could provide the information.

#### Eligible criteria

- Preterm babies (Babies delivered at gestational age less than 37 completed weeks).
- Preterm babies less than 24 h old (from time of delivery).
- Babies with no congenital malformations.

#### Temperature measurement

We documented admission temperatures for each baby and retrieved the lowest and highest daily recorded temperatures for each baby from their vital sign charts over the next 72 h of admission. In our setting, at least 4 sets of vital signs (including temperature) are typically taken and recorded within 24 h. Axillary temperature was measured using a digital thermometer (MT-101 model of UMEC^®^) which measures the temperature from 32 °C to 42.9 °C with an accuracy of ± 0.2 °C following standard procedures [22].

#### Quality control

Data collection was supervised by a senior pediatrician at each of the four sites. These pediatricians performed routine data quality checks. A second round of checks was conducted by a second pediatrician when all the data were pooled together.

### Variables

#### Outcome variable

Our outcome variable was hypothermia, which was classified as mild (36.0–36.4 °C), moderate (32.0–35.9 °C) or severe (< 32.0 °C) based on the World Health Organization (WHO) classification [22].

### Exposure variables

We identified previously published exposure variables in the literature and added a few biologically plausible factors that could cause hypothermia. For example, birth weight, gestational age and place of birth are well-known reported factors associated with hypothermia and documented in the literature [2, 23–25]. In addition, we considered other less reported local risk factors for example, preterm infants transported to hospitals using commercial vehicles. Our variable selection was performed before conducting any statistical analysis. Below is a list of variables we examined in our analysis:

#### The neonatal variables

Included age at admission, sex, admission weight, place of birth, estimated gestational age and active resuscitation at birth.

#### Obstetric variables

Place of delivery and presence of skilled birth attendants at delivery.

#### Maternal variables

Maternal age, parity, education, previous preterm delivery and spouse’s education.

#### Other variables

Mode of transportation to the newborn unit and period of admission.

### Data analysis

#### Data categorization

We categorized patients according to their temperature at admission into groups according to the World Health Organization (WHO) classification for hypothermia - mild, moderate, severe hypothermia and normal temperature. Preterm infants were also categorized into low-birth-weight infants > 1.5 kg to < 2.5 kg, very low birth weight > 1.0 kg to < 1.5 kg and extremely low birth weight < 1.0 kg. We also categorized these infants based on the place of delivery into inborn and outborn delivered babies. Inborn babies were infants delivered at our study hospitals, while outborn babies included those born outside of the study facilities, for example, those referred from other hospitals or those who were delivered at home. We considered additional categorization of the outborn deliveries into home delivery and outside hospital delivery. The education status of the parents was classified as formal education and defined as at least a primary school education; and no formal education. Maternal parity was classified as primiparous, defined as the index baby being the first delivery and multiparity was defined as having at least one delivery before the current child.

### Statistical analysis

Data analysis was conducted using STATA statistical software, version 17.1 (Stata Corp. 2020 Stata Statistical Software: Release 17. College Station, TX: Stata Corp LP). We performed summary statistics for the recruited cohorts and used clustered bar charts to describe the main outcome – preterm infant temperature. We then conducted a univariate analysis using Chi square test to compare categories and the Mann‒Whitney U test to compare group medians. A p-values < 0.05 indicated statistical significance in our univariate analysis.

For our primary analysis examining risk factors for moderate/ severe hypothermia at admission in preterm infants, we conducted a multivariate logistic regression. We selected variables for which p value was < 0.20 according to univariate analysis and included them in the maximal model. We cross-checked for collinearity among variables and computed two maximal models as we included variables that were collinear in separate models e.g. estimated gestational age and birth weight were collinear, while the place of delivery and skilled birth attendance also demonstrated collinearity. We performed backward elimination, dropping the least significant covariate until we had only significant variables in our model. For both models, we reported the adjusted odd ratios and their corresponding 95% confidence intervals to describe the strength of the association. Odds ratios were considered significant when the 95% CI did not cross 1.00.

## Results

Between August 2020 and July 2021, 933 preterm infants were recruited from the four study centers.

### Patient demographic characteristics

We recruited 477 (51.1%) male and 456 (48.9%) female infants. The median (interquartile range) estimated gestational age of our cohort was 33 weeks (30 weeks to 34 weeks). The median (interquartile range) weight was 1600 g (1300 g to 1900 g). In the cohort, 83 (8.9%) babies had extremely low birth weights while 279 (29.9%) of the babies had very low birth weights. There were 545 (58.5%) and 26 (2.8%) babies with low birth weights and normal weights respectively.

### Prevalence of hypothermia and variations across hospitals

Of the 933 preterm infants whose temperature was recorded at admission, approximately one quarter (*n* = 251, 26.9%) had a normal admission temperature; almost a third (*n* = 262, 28.1%) had mild hypothermia and less than half (*n* = 419, 44.9%) had moderate hypothermia. Only 1 (0.1%) baby presented with severe hypothermia.

#### Variation in hypothermia across hospitals

**Fig. 1:**
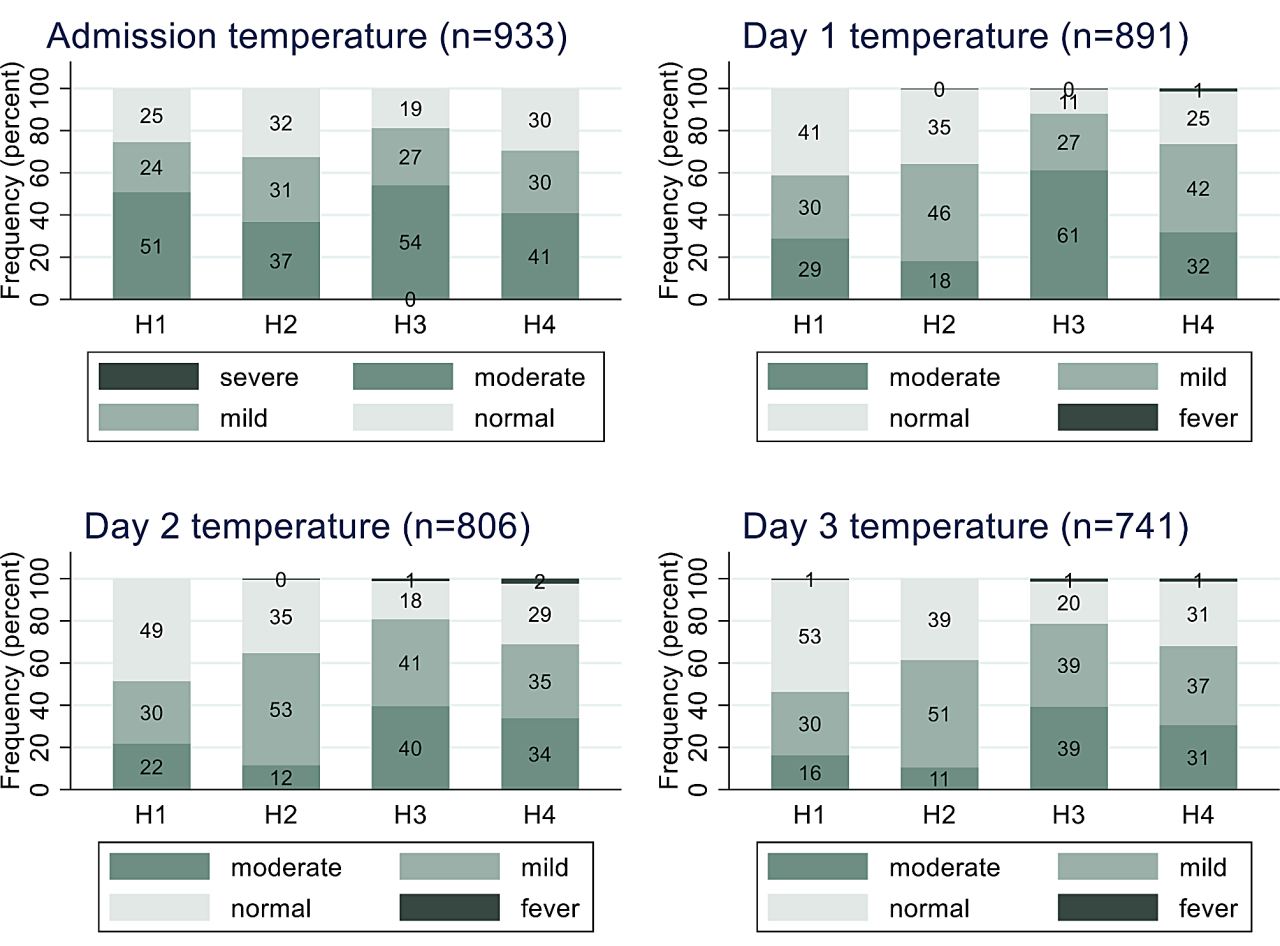
Temperature categories of babies at admission and lowest recorded temperature 3 days after admission

There was a marked variation in the prevalence of admission hypothermia across the hospitals. H3 had the highest prevalence with 81% of babies experiencing mild or moderate hypothermia at admission while H2 had the lowest prevalence (68%) (Fig. 1). Over the next 72 h of hospitalization, preterm infants still experienced hypothermia across all the hospitals with still H3 having the highest prevalence of hypothermia among all the admitted babies (Fig. 1). On Day 1, approximately 88% of babies in H3 had at least one episode of hypothermia and this proportion dropped to 81% and 78% on Days 2 and 3 respectively (Fig. 1). Of the centers, babies admitted in H1 had the best temperature control during the 72 h of monitoring.

### Newborn infant characteristics

The median age (± IQR) at admission and sex did not significantly differ between preterm infants with moderate/ severe hypothermia at admission and those without. Both groups differed significantly with regard to place of birth, presence of a skilled birth attendant at delivery and need for neonatal resuscitation at birth. A greater proportion of preterm infants with moderate/ severe hypothermia were born at home and were referred from other facilities (*p* = 0.001, Table 2) than those without such hypothermia. Similarly, a greater proportion of those with moderate/ severe hypothermia did not have a skilled birth attendant present at delivery (*p* = 0.029) and a high proportion were babies who required active resuscitation at birth (*p* = 0.001, Table 2).

**Table 2:** Maternal, obstetric, and newborn infant characteristics compared between preterm infants admitted with moderate/ severe hypothermia and those without (*n* = 933)

Variable	Hypothermia			
	Moderate/ severe hypothermia (%) *n* = 420	No moderate/ severe hypothermia (%) *n* = 513	*P*-value	Total (%) *n* = 933
**Age at admission (hours) Median ± IQR**	1 ± 2.0	1 ± 1.0	0.05	1 ± 2.0
**Gender**				
Male	224 (53.3)	253 (49.3)	0.22	477 (51.1)
Female	196 (46.7)	260 (50.7)		456 (48.9)
**Place of birth**				
Inborn	281 (67.1)	400 (78.3)		681 (73.2)
Outborn (outside hospital)	103 (24.6)	84 (16.4)		187 (20.1)
Outborn (Home)	35 (8.4)	27 (5.3)	**0.001**	62 (6.7)
**Skilled birth attendant present at delivery**				
Yes	390 (92.9)	493 (96.1)	**0.029**	883 (94.6)
No	30 (7.1)	20 (3.9)		50 (5.4)
**Required active neonatal resuscitation at birth**				
Yes	96 (22.9)	74 (14.4)	**0.001**	170 (18.2)
No	323 (77.1)	439 (85.6)		762 (81.8)
**Transportation mode to the neonatal unit**				
Brought from the labour ward	286 (68.1)	395 (77.3)	**0.003**	681 (73.2)
Commercial vehicle	80(19.1)	55(10.8)		135(14.5)
Personal vehicle	47 (11.2)	51 (10.0)		98 (10.5)
Ambulance	7 (1.7)	10 (2.0)		17 (1.8)
**Period of admission**				
Weekday	311 (74.1)	386 (75.2)	0.68	697 (74.7)
Weekend	109 (25.9)	127 (24.8)		236 (25.3)
**Previous history of preterm birth in the mother**				
Yes	44 (10.5)	49 (9.6)	0.63	93 (10.0)
No	375 (89.5)	464 (90.5)		839 (90.0)
**Weight Category**				
Normal	6 (1.4)	20 (3.9)	**< 0.001**	26 (2.8)
LBW	220 (52.4)	325 (63.5)		545 (58.5)
VLBW	135 (32.1)	144 (28.1)		279 (29.9)
ELBW	59(14.1)	24(4.5)		83(8.9)
**Maternal age (Median ± IQR)**	27 ± 10	28 ± 5	0.07	27 ± 9
**Estimated gestational age (Median ± IQR)**	32 ± 4	33 ± 3	**< 0.001**	
**Father’s educational status**				
Formally educated	338 (80.5)	429 (84.5)	0.112	767 (82.7)
No formal education	82 (19.5)	79 (15.6)		161 (17.4)
**Mother’s educational status**				
Formally educated	296 (70.5)	387 (75.9)	0.063	683 (73.4)
No formal education	124 (29.5)	123 (24.1)		247 (26.6)
**Maternal parity**				
Primiparous	139 (33.2)	160 (31.2)	0.52	299 (32.1)
Multiparous	280 (66.8)	353 (65.8)		633 (67.9)

ELBW: Extremely low birth weight, VLBW: Very low birth weight, LBW: Low birth weight

Inborn – Babies who are born within the study hospital, Outborn – Babies born in other hospitals and referred

Approximately 68.1% of the babies born in the hospitals and transferred to the neonatal unit had moderate/ severe hypothermia (Table 2). The relative proportion of babies who were transported to facilities using commercial transport was significantly greater in the moderate/ severe group than in the group without moderate/ severe hypothermia (19.1% versus 10.8%). Maternal age, parity and parental education status were similar between the groups (Table 2).

### Risk factors for moderate to severe hypothermia at admission

Preterm infants who were born by a skilled birth attendant (nurse, doctor or midwife) were 47% less likely to have moderate/ severe hypothermia at admission (Table 3- OR:0.53, 95% CI – 0.29 to 0.95). Birth weight was also a significant risk factor for moderate/ severe hypothermia. ELBW and VLBW infants were 8 and 3 times more likely to have moderate or severe hypothermia respectively at admission than preterm babies whose weights were ≥ 2500 g (Table 3). Each week’s increase in gestational age was associated with a 14% decrease in the risk of moderate/ severe hypothermia. Preterm babies born at home and at a pre-referral hospital had 1.9 fold and 1.6 fold greater odds of having moderate or severe hypothermia respectively than did those born at the study hospitals (Table 3). Those babies delivered with failure to initiate spontaneous respiration, who were actively resuscitated and thereby exposed, were also at increased risk of moderate/ severe hypothermia.

**Table 3:** Risk factors for moderate/ severe hypothermia at admission among preterm infants

Variable	Model 1 Adjusted OR (95% Confidence interval)	Model 2 Adjusted OR (95% Confidence interval)
**Skilled birth attendant present at the delivery**		
Yes	0.53 (0.29 to 0.95)	NA
No	Reference	
**Weight category**		
ELBW	8.11 (2.87 to 22.91)	NA
VLBW	3.08 (1.19 to 7.95)	NA
LBW	2.22 (0.87 t0 5.65	NA
Normal	Reference	
**Actively resuscitated at delivery**		
Yes	1.79 (1.27 to 2.53)	,
No	Reference	Reference
**Place of delivery**		
Outborn (Home)	NA	1.94 (1.13 to 3.33)
Outborn (outside hospital)	NA	1.64 (1.17 to 2.30)
Inborn		Reference
**EGA**	NA	0.86 (0.82 to 0.91)

OR- Odd ratios

## Discussion

In this study, we describe the occurrence of and risk factors for admission hypothermia in a cohort of preterm infants and examine the prevalence of hypothermia in the first 72 h after admission. This is one of the few Nigerian studies describing hypothermia at admission and in the first 72 h after admission; most studies focused on admission hypothermia. Our study revealed a high rate of admission hypothermia with an average incidence of 73.1% of all preterm infants having hypothermia at admission across the study facilities. These findings are similar to the 74.1%, 76.8% and 79.6% reported in studies from Korea, Ethiopia and Taiwan respectively [3, 10, 26]. In contrast, previous studies in northern Nigeria, Canada, and the United States of America reported lower prevalence rates of 42.5%, 36%, and 56.2% respectively [2, 12, 25] One possible explanation for the higher prevalence rate in our study compared to the other cited studies is the fact that our study included only preterm babies who are at higher risk of hypothermia. Even though the Canadian study focused on preterm neonates with a gestational age of no more than 33 weeks, the exclusion of babies born outside the facilities might explain the lower prevalence reported. Additionally, admission hypothermia prevalence of 88.2% reported from a study in China [9], is higher than that reported in the current study. This might be due to the inclusion of only preterm infants weighing less than 1,500 g in the study. The cold ambient temperature of China could also be a contributing factor.

The distribution of admission hypothermia (AH) in our study showed that the majority of the patients had either mild or moderate form with only one case of severe hypothermia among the cohorts which is consistent with the findings of Miller et al. and other workers [6, 25, 27, 28]. It could be that such low temperature is not compatible with life in majority of preterm neonates with limited adaptation to thermal stress.

In the preset study, a significant number of babies delivered in the facilities had hypothermia at admission. This finding suggests, perhaps, that more focus should be given to the ambient temperatures in delivery rooms at various facilities. This might also be a reflection of poor intrafacility transfer systems from delivery rooms to newborn units. This calls for stringent measures for temperature control in these delivery suites and the neonatal units.

In the present study, hypothermia occurred frequently and persisted in some babies up to 72 h after admission to the neonatal units. We focused on the first 72 h post admission as the stabilization of babies and the majority of procedures take place during that period which could result in hypothermia. In our study, more than 70% of the premature neonates were hypothermic 24 h later, and after 72 h, a significant proportion of the neonates were still hypothermic. Our finding was higher than the 55% reported by Demstre et al. [3], after 12 h of hospitalization in a study in Ethiopia. Several factors might have contributed to the hypothermia of the study subjects even after admission to the neonatal units. These might include poor practice of kangaroo mother care, limited number of incubators, inadequate radiant warmers and low nurse-to-patient in the participating hospitals. Additionally, the newborn units have no wall thermometers available for measuring ambient temperature thereby making it difficult to monitor and ensure optimal thermoneutral temperatures within the units. We observed variability across the study sites in their capacity to maintain the babies’ temperature within normal in the 72 h following admission with one center performing fairly better than others. This might be a reflection of differences in the availability of human and material resources across the facilities.

In this study the associations between admission hypothermia and gestational age, birth weight, place of delivery, resuscitation at birth and mode of transportation to the neonatal units were significant. The relationship between gestational maturity and hypothermia noted in our study is similar to the findings of an East African meta-analysis, the Moi Teaching Hospital Study in Kenya and studies from the Netherlands [27, 29–31] The temperature at admission was found to be inversely related to gestational age; the younger the gestational age was, the greater the risk of hypothermia. Therefore, our study, aligns with the documented inverse relationship between gestational age and the risk of hypothermia [3]. The risk of hypothermia among preterm neonates is a function of their physiology which limits their thermogenic capacity compared to that of term infants [30]. In contrast, a Korean neonatal network study indicated that the effect of gestational age on temperature control was limited but that birth weight was more strongly associated with admission hypothermia [26]. This finding might be related to the non-inclusion of all categories of preterm babies in the Korean study which focused only on very low birth weight preterm babies weighing less than 1,500 g. Small for gestational age (SGA) babies were also excluded from the study.

Like the findings of Mank et al. [27], Lee et al. [26], and Laptook et al. [32] we report an association between birth weight and hypothermia in the present study. We found that birth weight was an important predictor of admission hypothermia in ELBW infants. This finding was also similar to that of a previous study in Kenya [31].

Another important factor that could be associated with hypothermia at admission is the mode of transportation of babies to the neonatal units. In the present study, this was found to be an important risk factor for admission hypothermia similar to the findings of a United States of America study in which hypothermia was found in up to 52% of neonatal admissions [33] In the latter study, following a quality improvement effort implemented to target the interfacility transport of VLBW and ELBW preterm infants, the incidence decreased from 52 to 17%. Furthermore, Glenn et al. and McNellis et al. in their different studies showed that it is feasible to reduce admission hypothermia significantly during neonatal transport by taking appropriate measures [33, 34]. Conversely, a study from southwestern Nigeria revealed that 35.1% of neonates transferred to a referral hospital were hypothermic [35]. These findings are much lower than the results from our study. The observed differences (72% versus 35.1%) could be because the southwestern Nigerian study included a heterogeneous population of preterm and term babies whereas our study included only preterm infants. Another explanation might be that some of the babies were in a skin-to-skin position during transportation while none of the babies in our study were in skin-to skin position during transport. In our study, babies transported to newborn units by commercial vehicles had an increased risk of hypothermia in contrast to the findings of a survey in a Nigerian study by Abdulraheem et al. in which the majority of the babies were transported by commercial vehicles but more than a quarter of the transported babies received prolonged skin-to-skin contact during transfer [20]. The finding in the current study that none of the babies were transported in the KMC position might be a reflection of a low level of awareness of this effective, low cost and easily adaptable intervention while transporting small infants in our community. This could also indicate that the babies were not stabilized before transferring to our facilities. Initial stabilization of neonatal patients before interfacility transport has been suggested to improve outcomes rather than the common practice of “scoop and run” approach [2, 23, 30].

Neonates delivered at home and hospitals outside of the study facilities were more likely to be hypothermic than neonates born at our study health centers similar to the findings of an East African study [36]. This might be because the delivery of such babies was unlikely to be attended by skilled personnel whose presence at birth was found to be protective against the development of hypothermia in our study. This might also be due to the poor transport system available for these babies to move from the site of birth to our newborn units. Furthermore, this might also be because mothers who delivered at home were more likely to engage in suboptimal thermal care practices, as reported in a study from Uganda [37]. These mothers, for instance, are more likely to bathe their babies soon after birth, a practice that has been shown to result in a substantial drop in the neonatal temperatures [38].

Another variable that showed significant association with hypothermia in our study was neonatal resuscitation. Babies that failed to initiate spontaneous respiration at birth who received cardiopulmonary resuscitation to help them breath were more likely to be hypothermic than were those who were not, a finding similar to reports from other studies [6, 24, 39]. Keeping infants sufficiently warm during resuscitation might be difficult due to inadequate number of radiant warmers at the various hospitals. This might have resulted in the resuscitation of newborns on babies’ cots or on other surfaces outside a radiant warmer and the resultant development of hypothermia as babies were exposed during such activities.

## Conclusion

The prevalence of point of admission hypothermia was high within this study context. Gestational age, admission/ birth weight, active resuscitation at birth, the presence of skilled birth attendants at delivery and poor referral transport are significant factors affecting the risk of developing hypothermia in preterm infants. The prevalence of hypothermia while in care is also high and this has important implications for patient safety and quality of patient care. Referral services for preterm infants need to be developed while hospitals need to be better equipped to maintain optimal temperatures for sick preterm babies. Increasing awareness about the prevalence and the possible risk factors associated with hypothermia may motivate the significant reduction of hypothermia and thereby improve the outcome of preterm babies.

### Strengths and limitations

A strength of this study is that it was a multicenter study that included a large cohort of preterm infants in neonatal units of tertiary health facilities across the three geopolitical zones of northern Nigeria providing a good representation of the findings. Our study not only assessed a single point admission temperature, but also included the temperature over the first 72 h after admission in addition to associated risk factors thereby providing a holistic picture of temperature control over a longer time window at various sites.

A limitation of our study is the non-availability of data on interventions used at referring hospitals, during neonate transport and at the study centers to minimize heat loss. Additionally, we did not know the temperatures of the delivery rooms of each study center or the place of birth of babies born outside the study centers.

## Data Availability

The data used in this study are available from the corresponding author upon reasonable request.
